# Advanced glycation end products (AGEs) increase renal lipid accumulation: a pathogenic factor of diabetic nephropathy (DN)

**DOI:** 10.1186/s12944-017-0522-6

**Published:** 2017-06-28

**Authors:** Yang Yuan, Hong Sun, Zilin Sun

**Affiliations:** 1 Department of Endocrinology, Affiliated Zhongda Hospital of Southeast University, No. 87 DingJiaQiao Road, Nanjing, 210009 People’s Republic of China; 2 Department of Endocrinology and Metabolism, The first Affiliated Hospital of Soochow University, 188 shizi street, suzhou, 215006 jiangsu China

**Keywords:** Nε-(carboxymethyl) lysine (CML), 3-hydroxy-3-methylglutaryl coenzyme A reductase (HMG-CoAR), LDL receptor (LDLr), Sterol regulatory element binding protein-2 (SREBP-2), SREBP cleavage-activating protein (SCAP), Diabetic nephropathy (DN)

## Abstract

**Background:**

Advanced glycation end products (AGEs) are pathogenic factors of diabetic nephropathy (DN), causing renal damage in various ways. The aim of this study is to investigate the ectopic lipid accumulation caused by AGEs in human renal tubular epithelial cell line (HK-2) cells and the kidney of type 2 diabetic rats.

**Methods:**

In vivo study, diabetes was induced in male Sprague–Dawley rats through intraperitoneal injection of high-fat/high-sucrose diet and low-dose streptozocin (STZ). Two weeks after STZ injection, the diabetic rats were randomly divided into two groups, namely, untreated diabetic and Aminoguanidine Hydrochloride (AG, an AGEs formation inhibitor)-treated (100 mg/Kg/day, i.g., for 8 weeks) group. In vitro study, according to the different treatments, HK-2 were divided into 6 groups. Intracellular cholesterol content was assessed by Oil Red O staining and cholesterol enzymatic assay. Expression of mRNA and protein of molecules controlling cholesterol homeostasis in the treated cells was examined by real-time quantitative PCR and western blotting, respectively. SREBP cleavage-activating protein (SCAP) translocation was detected by confocal microscopy.

**Results:**

Here we found Nε-(carboxymethyl) lysine (CML, a member of the AGEs family) increased Oil Red O staining and intracellular cholesterol ester (CE) in HK-2 cells; Anti-RAGE (AGEs receptor) reduced lipid droplets and the CE level. A strong staining of Oil Red O was also found in the renal tubules of the diabetic rats, which could be alleviated by AG. CML upregulated both mRNA and protein expression of 3-hydroxy-3-methylglutaryl coenzyme A reductase (HMG-CoAR), LDL receptor (LDLr), sterol regulatory element binding protein-2 (SREBP-2) and SCAP, which were inhibited by anti-RAGE. The upregulation of these molecules in the kidney of the diabetic rats was also ameliorated by AG. Furthermore, AG reduced serum and renal CML deposition, and improved urine protein and u-NGAL in type 2 diabetic rats.

**Conclusions:**

Overall, these results suggest that CML caused DN might be via disturbing the intracellular feedback regulation of cholesterol. Inhibition of CML-induced lipid accumulation might be a potential renoprotective role in the progression of DN.

## Background

Type 2 diabetes mellitus (T2DM) is one of the world’s most common chronic metabolic disorders of multiple aetiologies. The World Health Organization (WHO) predicts that the number of people with T2DM will double to at least 350 million worldwide by 2030 [1]. The characteristic of T2DM is chronic hyperglycemia, accompanied by an accelerated rate of advanced glycation end products (AGEs) formation. AGEs derived from reducing sugars reaction non-enzymatically with amino groups of protein play an important role in the pathogenesis of diabetic complications [2]. Nε-(carboxymethyl) lysine (CML) is one of the major AGEs in vivo [3], and its level increases in serum and organs (such as kidney) of diabetic patients [4–7]. The increased circulating CML and accumulation of CML in tissues have been recognized as a critical step in the pathogenesis of insulin resistance, dyslipidaemia, and diabetic nephropathy (DN) [8, 9], however, the definite mechanisms are still unknown.

DN is one of the most serious microvascular complications of diabetes, and the major cause of end-stage renal disease (ESRD) worldwide. The pathophysiologic changes in DN include hyperfiltration and microalbuminuria followed by worsening of renal functions associated with cellular and extracellular derangements in both the glomerular and the tubulointerstitial compartments [10]. Recent type 2 diabetic human and experimental studies have associated ectopic lipid accumulation in the kidney (fatty kidney) [11, 12]. Multiple enzymes, carrier proteins, and lipoprotein receptors are involved in fatty kidney foam cell formation. Low density lipoprotein receptor (LDLr) is the channel for uptaking cholesterol [13] and 3-hydroxy-3-methylglutaryl coenzyme A reductase (HMG-CoAR) is the key enzyme for cholesterol synthesis [14]. These two proteins are regulated by sterol regulatory element binding protein-2 (SREBP-2). SREBP cleavage-activating protein (SCAP) has been identified as a cholesterol sensor and chaperone of SREBP-2. When cells demand cholesterol, SCAP shuttles SREBP-2 from the endoplasmic reticulum (ER) to the Golgi, where SREBP-2 are cleaved by two proteases (site 1 and site 2 proteases). The cleaved SREBP-2 N-terminal fragment enters into the nucleus, binds to the sterol-regulatory elements in the HMG-CoAR and LDLr promoters, and upregulates their transcription, resulting in increases of cholesterol uptake and synthesis. However, when the intracellular concentration of cholesterol is high, the SCAP-SREBP complex is retained in the ER, and doesn’t perform the subsequent regulation. This feedback regulation mediated by SCAP can prevent overloading of intracellular cholesterol under physiological condition [15–17].

Our previous study has already showed lipid accumulation in the kidney of type 2 diabetic rats [18]. Therefore, the current study is undertaken to provide an explanation for the above phenomenon by studying the effects of CML on LDLr-mediated cholesterol uptake and HMG-CoAR-mediated cholesterol synthesis in human renal tubular epithelial cell line (HK-2) and the kidney of type 2 diabetic rat model.

## Methods

### Animal experimental design

Male Sprague–Dawley rats weighing 150-170 g were purchased from shanghai SIPPRBK laboratory animals ltd (Shanghai, China). After 1 week adaptation, rats were given high fat/sucrose diet (67% standard chaw, 10% lard, 20% sugar, 2.5% cholesterol and 0.5% sodium cholate). Four weeks later, the rats were injected with 35 mg/kg STZ (dissolved in 0.01 mol/L citrate buffer, pH 4.5) intraperitoneally. After 72 h, only rats with a non-fasting blood glucose of ≥16.7 mmol/l were considered diabetic and selected for additional studies [19]. Two weeks later, the rats were divided into 2 groups: DM group and DM + AG group (intragastric administration of Aminoguanidine Hydrochloride, 100 mg/kg, dissolved in water) [20]. Twenty four hour urine of rats was collected in individual metabolic cages to measure urine protein and urinary neutrophil gelatinase-associated lipocalin (u-NGAL) level. At the end of the 8th week, the rats were fasting overnight, then sacrificed .The blood was collected to separate the serum used for test blood urea nitrogen (BUN), creatinine (Cr), total triglyceride (TG), total cholesterol (TC), high density lipoprotein (HDL), LDL, CML. Part of the kidney was fixed in 10% neutral formalin and embedded in paraffin for immunohistochemical staining, periodic acid Schiff (PAS) staining, and periodic acid-silver metheramine (PASM) staining. Part of the kidney was fixed in 4% paraformaldehyde, then dehydrated and embedded in OCT for Oil Red O staining. The left tissue was immediately stored at −80 °C for quantitative RT-PCR and Western blot.

### Biochemical assay

Serum BUN, Cr, TG, TC, HDL and LDL were determined using fully automatic biochemical analyzer in Zhongda Hospital. Serum CML was determined by HPLC-MS/MS analyzer. Twenty four hour urine protein was measured by Coomassie brilliant blue protein assay (Jiancheng Bioengineering Institute, Nanjing, Jiangsu). U-NGAL was measured using ELISA method provided by USCN (Wuhan, China).

### Renal histology

Sequential paraffin-embedded tissue sections from the renal cortex were cut. Cross sections (3 um) were placed on gelatin-coated slides and disposed of immunohistochemical staining for CML, PAS and PASM staining.

### Cell culture

HK-2 cells (a gift from Dr. BC Liu) were cultured with Dulbecco’s Modified Eagle’s Medium/Ham’s Nutrient mixtureF-12 (DMEM-F12) containing 10% fetal bovine serum. All experiments were carried out in serum-free DMEM-F12 medium containing 0.2% bovine serum albumin (BSA), 100 U/ml penicillin and 100 μg/ml streptomycin. Reagents for cell culture were obtained from HyClone (Logan, Utah, USA). CML, which was produced by organic synthesis (no material of animal or human origin is used), was obtained from Santa Cruz (Delaware Avenue, USA). Low density lipoprotein (LDL) was purchased from Yiyuan Biotechnologies (Guanzhou, China). Anti-RAGE (receptor of AGEs), which was used to block CML-RAGE pathway, was obtained from R&D Systems (Minneapolis, MN, USA).

### Observation of lipid accumulation

The lipid accumulation in HK-2 cells and kidney of type 2 diabetic rats was evaluated by Oil Red O staining. Briefly, samples were fixed with 4% paraformaldehyde and then stained with Oil Red O for 30 min. Then, the samples were counterstained with hematoxylin for 5 min. Results were examined by light microscopy.

### Quantitative measurement of intracellular cholesterol

HK-2 cells in six-well plates were cultured for 24 h in different experimental conditions. Then cells were washed twice in PBS, total cholesterol (TC) and free cholesterol (FC) content were measured by enzymatic assays (Applygen Technologies Inc., Beijing, China). The concentration of cholesterol ester (CE) was calculated using TC minus FC.

### Quantitative RT-PCR

Total RNAs were isolated from HK-2 cells or renal homogenates of type 2 diabetic rats with TRIzol reagent (Invitrogen, USA). Then, RNA (1 μg) was used as a template for RT with a High Capacity cDNA RT Kit from ABI (Applied Biosystems, Warrington, UK). Real-time RT-PCR was performed in an ABI 7000 Sequence Detection System using SYBR Green dye according to the manufacturer’s protocol (Applied Biosystems, Warrington, UK). All PCR primers (Jierui Biotechnology, Shanghai, China) are shown in Table 1.

**Table 1:** The primers for real-time RT-PCR

Gene	Primers
HMG-CoAR	5'- TACCATGTCAGGGGTACGTC −3' sense
	5'- CAAGCCTAGAGACATAATCATC −3' antisense
LDLr	5'-CCAAATGATGCCACTTCCC −3' sense
	5'- ATCCCATCCCAACACACAC −3' antisense
SREBP-2	5'- CCCTTCAGTGCAACGGTCATTCAC −3' sense
	5'- TGCCATTGGCCGTTTGTGTC −3' antisense
SCAP	5'- GGCATCAAGTTCTACTCCATTC-3' sense
	5'- CCAGTTGGAATGCTCGGGAC-3' antisense
GAPDH	5'- TGTTGCCATCAACGACCCCTT −3' sense
	5'- CTCCACGACATACTCAGCA −3' antisense

### Western blot

Protein was separated on 10% SDS-PAGE gel. Polyvinylidene fluoride membrane (Millipore Corporation, Bedford, MA, USA) was used for transfer and then blocked for 1 h at room temperature with 5% bovine serum albumin in Tris-buffered saline containing 0.05% Tween 20 (TBST). Subsequently, blots were washed and incubated overnight at 4 °C in TBST containing 5% bovine serum albumin with a 1:1000 dilution of SCAP, SREBP-2, LDLr, and HMG CoAR antibody and β-actin antibody (Abcam, Cambridge, UK). Membranes were washed three times with TBST, incubated with a secondary antibody (1:5000 dilutions in TBST containing 1% bovine serum albumin; Santa Cruz Biotechnology) for 1 h at room temperature and then washed three times with TBST. After the chemiluminescence reaction (Pierce, Rockford, IL, USA), bands were detected by exposing blots to X-ray films for the appropriate time period. For quantitative analysis, bands were detected and evaluated densitometrically with LabWorks software (UVP Laboratory Products, Upland, CA, USA), normalised for β-actin density.

### Plasmid constructions

A green fluorescent protein (GFP)-SCAP expression construct was made by ligating human SCAP cDNA into the BstE-XbaI sites of the pEGFP-C1 vector (Genechem Co. Ltd., Shanghai, China).

### Confocal microscopy

HK-2 cells were plated on chamber slides and incubated in growth medium for 24 h. Cells were subsequently transfected with pEGFP-SCAP using Effectene Transfection Reagent (Invitrogen, Paisley, UK) according to the manufacturer’s protocol. After being treated in different experimental conditions for 24 h, the transfected cells were fixed in 5% formalin solution for 30 min, permeablized with 0.25% of Triton X-100 for 15 min, and stained with mouse anti-Golgin-97 antibody (Molecular Probes, Inc., Eugene, USA) for 2 h at room temperature. After washing, the cells were further stained by a secondary fluorescent antibody (goat anti-mouse Alexa Fluor 594) for 1 h. Results were examined by confocal microscopy using a Zeiss LSM 510 Meta (Carl Zeiss, Hertfordshire, UK).

### Statistics

All experiments were repeated at least three times. In all experiments, data were expressed as mean ± SD and analysed using SPSS 18.0 for Windows. Means of every pair of data sets were determined with Student’s *t*-test. *P* < 0.05 was considered to be statistically significant.

## Results

### Inhibiting CML formation reduced renal lipid accumulation in type 2 diabetic rats

**Fig. 1:**
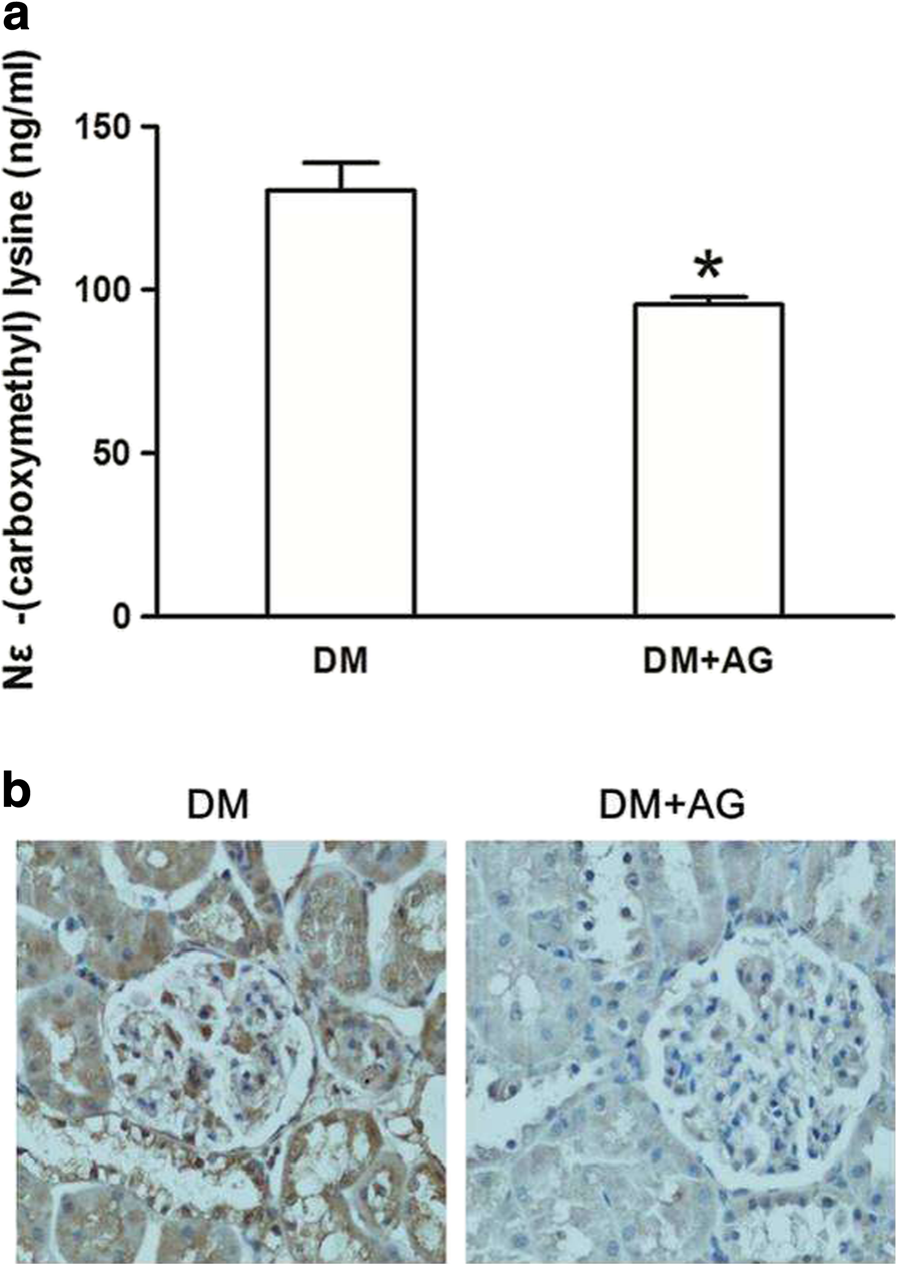
The production of Nε -(carboxymethyl) lysine (CML) in the serum or kidney of type 2 diabetic rats with or without Aminoguanidine Hydrochloride (AG) intragastric administration. **a** The level of CML in the serum of type 2 diabetic rats was checked by HPLC-MS analyzer. Results represent the mean ± SD (*n* = 6). *P < 0.05, DM + AG vs. DM. **b** The distribution of CML in the kidney of type 2 diabetic rats was checked by immunohistochemical staining

**Fig. 2:**
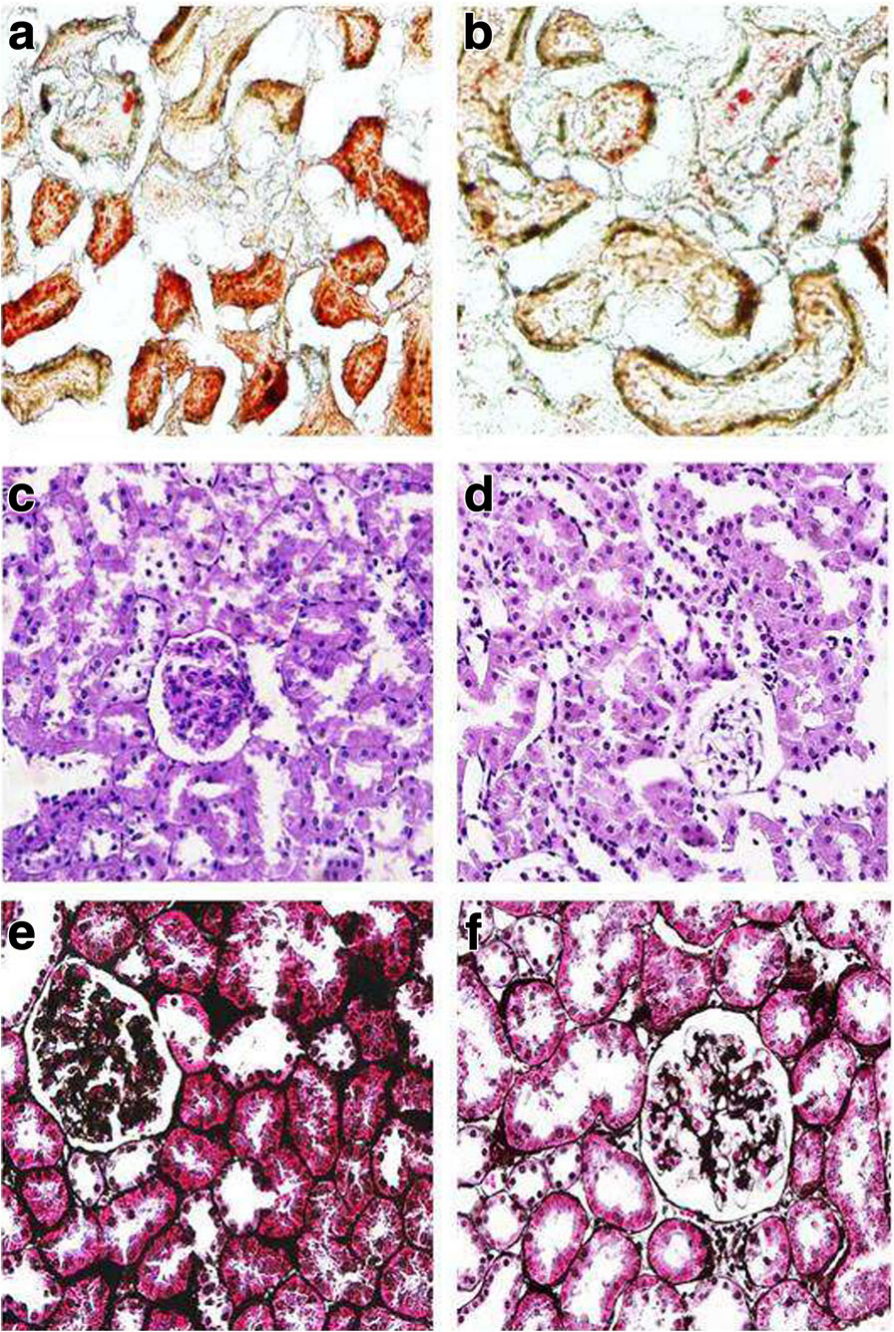
Oil Red O staining staining. (×200, **a** and **b**); PAS staining. (×400, **c** and **d**); PASM staining. (×400, **e** and **f**). DM: **a**, **c**, and **e**; DM + AG: **b**, **d**, and **f**

We used the high fat/ sucrose diet and STZ-induced type 2 diabetic rats for the in vivo study. HPLC-MS/MS analysis showed there was an increase of serum level of CML in diabetic rats (Fig. 1a); in addition, significant deposition of CML was found in the diabetic renal tubules, glomerulus, mesangium, basement membrane and interstitium by immumohistochemical staining (Fig. 1b), and this is consistent with other researches [7, 21]. However, AG treatment reduced CML both in the serum and the kidney of diabetic rats (Fig. 1a and b). Oil Red O staining showed lipid droplets were accumulated in the kidney of the diabetic rats. Interestingly, the lipid accumulation in the renal tubules was more obvious than that in the renal glomeruli; AG treatment alleviated renal lipid accumulation (Fig. 2a and b). These results suggest a strong association between CML and enhanced lipid accumulation in the diabetic kidney.

### Blocking CML-RAGE pathway ameliorated CML induced lipid deposition in HK-2 cells

**Fig. 3:**
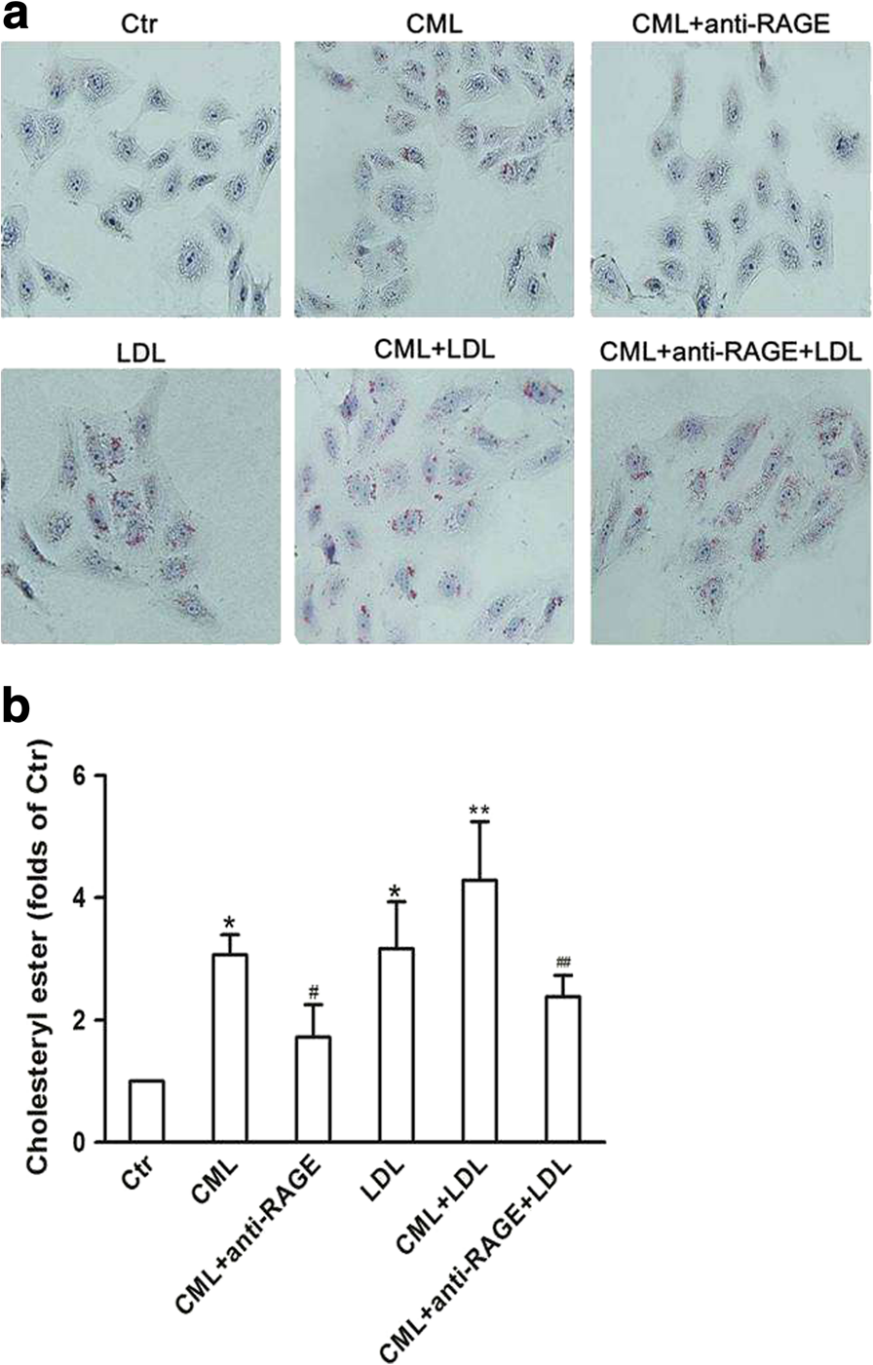
Visualization of LDL uptake and lipid droplets in human renal tubular epithelial cell line (HK-2) after Nε -(carboxymethyl) lysine (CML) treatment. HK-2 cells were incubated for 24 h in experimental medium, or medium containing 50 μg/ml CML or 200 μg/ml LDL, or 50 μg/ml CML plus 10 μg/ml anti-RAGE, or 50 μg/ml CML plus 200 μg/ml LDL, or 50 μg/ml CML plus 200 μg/ml LDL and 10 μg/ml anti-RAGE. **a** Cells were examined for lipid inclusions by Oil Red O staining. The results are typical of those observed in 3 separate experiments (×200). **b** The concentration of cholesterol ester in HK-2cellswas measured as described in Materials and Methods. Values are mean ± SD of duplicate wells from 3 experiments. *P < 0.05 vs. Ctr; **P < 0.05 vs. LDL group; ^#^ *P* < 0.05 vs. CML group; ^##^ *P* < 0.05 vs. CML+ LDL group

By in vitro study, we demonstrated that increased lipid droplets in HK-2 cells in the presence of native LDL or CML; more significant lipid droplets were found in cells treated with both LDL and CML; and anti-RAGE reduced the lipid droplets accumulation in the CML-treated cells with the absence or presence of a high concentration of LDL (Fig. 3a). Further quantitative analysis of intracellular cholesterol ester confirmed the results from Oil Red O staining (Fig. 3b). These suggest that CML increases cholesterol content in HK-2 cells through the CML-RAGE pathway.

### Inhibiting CML formation improved renal morphology and function in type 2 diabetic rats

**Fig. 4:**
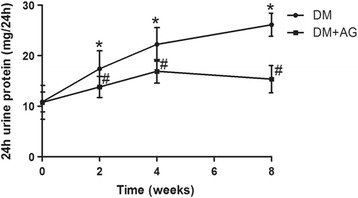
The 24-h urine protein of type 2 diabetic rats with or without Aminoguanidine Hydrochloride (AG) intragastric administration. 24-h urine protein was measured by Coomassie brilliant blue protein assay. Results represent the mean ± SD (*n* = 6). *P < 0.05, DM + AG vs. DM; ^#^ *P* < 0.05, DM group, 8w vs. 4w, 4w vs. 2w, 2w vs. 0w

**Fig. 5:**
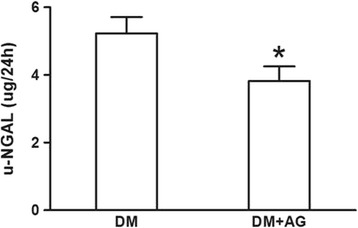
The urinary neutrophil gelatinase-associated lipocalin (u-NGAL) level of type 2 diabetic rats with or without Aminoguanidine Hydrochloride (AG) intragastric administration. U-NGAL was measured by ELISA kits. Results represent the mean ± SD (*n* = 6). *P < 0.05, DM + AG vs. DM

The level of BUN, Cr, TG, TC, HDL and LDL was markedly higher in diabetic rats (data were showed in previous study) [18]; AG treatment reduced serum Cr (67.00 ± 14.35 vs. 39.00 ± 7.84 μmol/l, *P* < 0.05); but the level of serum BUN was not alleviated by AG treatment (9.26 ± 1.44 vs. 10.56 ± 1.23 μmol/l, *P* > 0.05); AG had no influence on the level of serum lipid (date not show) as well. 24 h urine of rats was collected in individual metabolic cages at the time when before AG treatment and AG treated for 2 weeks, 4 weeks and 8 weeks. 24 h urine protein of the diabetic group was significantly increased, and it continued to elevate with the progression of diabetes, however, 4 weeks and 8 weeks-treatment of AG improved this alteration (Fig. 4). AG treated for 8 weeks also reduced the level of u-NGAL (Fig. 5). PAS staining (Fig. 2c and d) and PASM (Fig. 2e and f) staining showed mesangial expansion in the renal glomeruli and basement membrane thickness both in the glomeruli and tubules of diabetic rats, which could be alleviated by AG treatment. The data above suggest that inhibiting CML formation could improve the renal morphology and function, this may be associated with the reduction of CML-induced lipid accumulation in the kidney.

### Inhibiting CML formation reduced gene and protein expression of HMG-CoAR, LDLr, SREBP-2 and SCAP in the kidney of type 2 diabetic rats

**Fig. 6:**
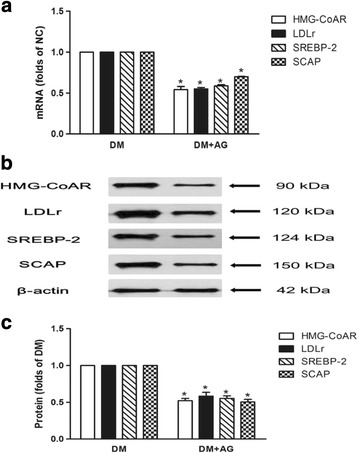
Effects of AG on mRNA and protein expression of HMG-CoAR, LDLr, SREBP-2 and SCAP. The mRNA levels were determined for real-time RT-PCR as described in Materials and Methods. GAPDH served as a reference gene. Results represent the mean ± SD from 3 experiments (*n* = 6) (**a**). The protein levels were examined by Western blotting (**b**). The histogram represents mean ± SD of the densitometric scans for proteins from 3 experiments (*n* = 6), normalized by comparison with β-actin and expressed as a percentage of control (**c**). *P < 0.05, DM + AG vs. DM

To investigate potential mechanisms of the phenomena, we evaluated the effect of AG on the gene and protein expression of HMG-CoAR, LDLr, SREBP-2 and SCAP in the kidney of diabetic rats. We found that AG downregulated both the mRNA and protein levels of HMG-CoAR, LDLr, SREBP-2 and SCAP (Fig. 6a, b and c ).

### Blocking CML-RAGE pathway downregulated CML induced gene and protein upregulation of HMG-CoAR, LDLr, SREBP-2 and SCAP in HK-2 cells

**Fig. 7:**
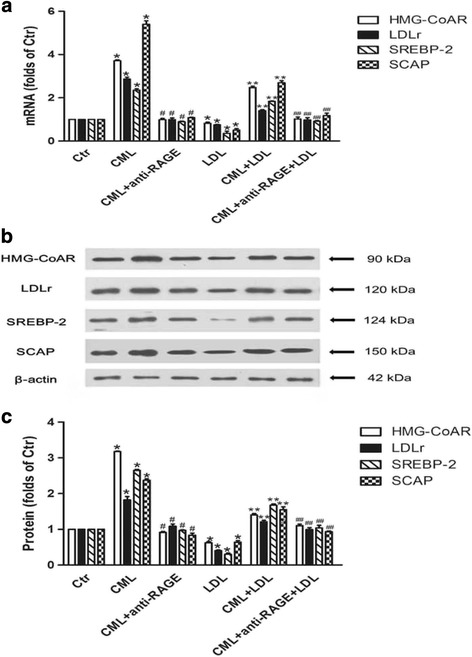
Effects of CML on the mRNA and protein expression of HMG-CoAR, LDLr, SREBP-2 and SCAP in HK-2 cells. HK-2 cells were incubated for 24 h in experimental medium, or medium containing 50 μg/ml CML or 200 μg/ml LDL, or 50 μg/ml CML plus 10 μg/ml anti-RAGE, or 50 μg/ml CML plus 200 μg/ml LDL, or 50 μg/ml CML plus 200 μg/ml LDL and 10 μg/ml anti-RAGE. The mRNA levels were determined for real-time RT-PCR as described in Materials and Methods. GAPDH served as a reference gene. Results represent the mean ± SD from 3 experiments (**a**). The protein level was examined by Western blot (**b**). The histogram represents means ± SD of the densitometric scans for proteins from 3 experiments, normalized by comparison with β-actin and expressed as a percentage of control (**c**). *P < 0.05 vs. Ctr; **P < 0.05 vs. LDL group; #*P* < 0.05 vs. CML group; ##*P* < 0.05 vs. CML+ LDL group

In vitro study showed that native LDL significantly inhibited HMG-CoAR, LDLr, SREBP-2 and SCAP gene and protein expression in HK-2 cells. However, CML increased the mRNA and protein levels of HMG-CoAR, LDLr, SREBP-2 and SCAP in the absence or presence of a high concentration of native LDL, and these could be inbibited by anti-RAGE (Fig. 7a, b and c).

### Blocking CML-RAGE pathway attenuated CML induced SCAP translocation from ER to the Golgi in HK-2 cells

**Fig. 8:**
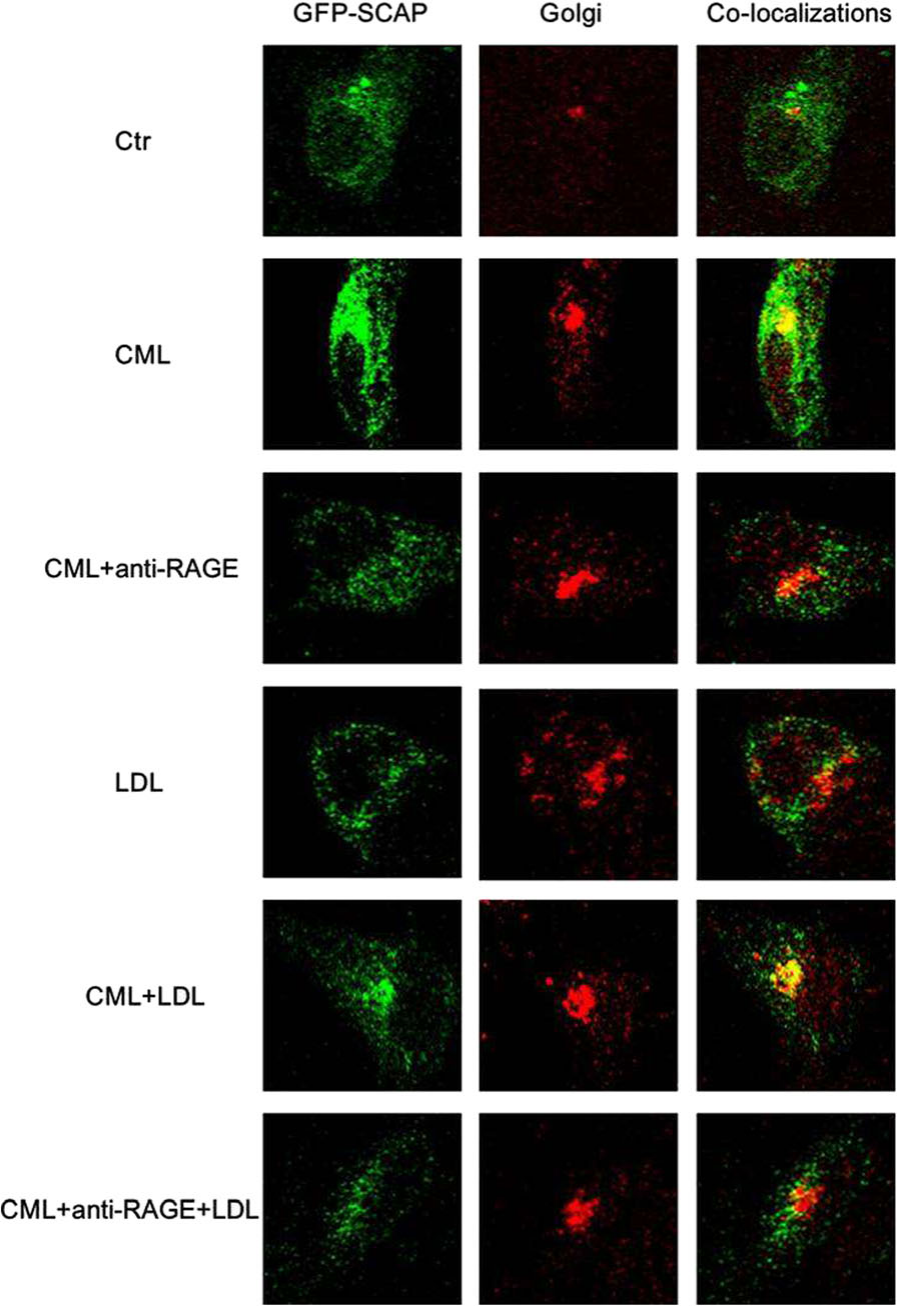
Effect of CML on protein translocation of pGFP-SCAP from the ER to the Golgi in HK-2 cells. Transiently transfected HK-2 cells were cultured in experimental medium, or medium containing 50 μg/ml CML or 200 μg/ml LDL, or 50 μg/ml CML plus 10 μg/ml anti-RAGE, or 50 μg/ml CML plus 200 μg/ml LDL, or 50 μg/ml CML plus 200 μg/ml LDL and 10 μg/ml anti-RAGE. The translocation of SCAP from the ER to the Golgi was investigated using confocal microscopy after staining with anti-Golgin antibody, as described in Materials and Methods

Using confocal microscopy, we investigated SCAP translocation between the ER and the Golgi in HK-2 cells. We found that LDL loading reduced SCAP accumulation in the Golgi, and interestingly, exposure to CML enhanced the localization of SCAP to the Golgi even in the presence of native LDL loading. However, anti-RAGE could inhibit CML induced SCAP transfer in HK-2 cells (Fig. 8).

## Discussion

Dysregulation of triglycerides in kidney has been elucidated in many studies, but the contribution of cholesterol in DN seems to have been neglected [11, 22–27]. We have already showed abnormal cholesterol metabolism in the kidney of type 2 diabetic rats in our previous study, and in this current study, we intend to explain the mechanisms for the cholesterol accumulation in the diabetic kidney. Katrien H.J. Gaens et al. demonstrated that hepatic steatosis is associated with CML deposition in the liver [28]. Since CML can affect enzymatic activity, modify protein, and alter immunogenicity [2], we suppose that CML may be a causative factor of renal cholesterol accumulation in T2DM.

In vivo study, we built the type 2 diabetic rat model. The rat model was induced by fed western diet and introperitoneal injection with STZ. Here, we didn’t use the spontaneous diabetes rodents for excluding congenital dyslipidemia. One group of the diabetic rats was given AG by gavage. AG is a powerful blocker of the AGEs pathway, though it has been largely supplanted in the clinical arena by other AGEs formation inhibitors [29], cross-link breakers [30, 31], and receptor antagonists [32]. Nevertheless, this compound remains a useful tool with which to assess the biological relevance of AGEs in vivo context. Our results showed significantly increased serum and renal tissue levels of CML in the diabetic rats, suggesting that systemic and local renal increasing CML were successfully induced in the rats. Oil Red O staining showed that lipid droplets accumulation in the kidney of the diabetic rats, especially in the renal tubules, and this was alleviated by AG, suggesting a strong association between CML and enhanced lipid accumulation in the kidney. Since the tubules expose to large quantities of CML, they are potential to be the most seriously part directly injured by CML [33].

To prove the results from in vivo study, we demonstrated that CML increased cholesterol accumulation in HK-2 cells even in the presence of a high concentration of LDL. In addition, we used anti-RAGE blocking the CML-RAGE pathway to definite the function of CML in causing intracellular cholesterol accumulation.

Next, we studied the SCAP-SREBP-2-LDLr/HMG-CoAR pathway to explore potential mechanisms of accelerated lipid accumulation induced by CML. Results showed that AG downregulated mRNA and protein expression of HMG-CoAR, LDLr, SREBP-2, and SCAP in the kidney of type 2 diabetic rats. In vitro study showed CML increased HMG-CoAR, LDLr, SREBP-2, and SCAP mRNA and protein expression, and enhanced the localization of SCAP to the Golgi in the absence or presence of native LDL loading, which further supported our in vivo findings. Here, native LDL was used to make excessive cholesterol loading, and active the intracellular cholesterol feedback regulation, displaying lower expression of LDLr and HMG-CoAR, and reduced SCAP transfer to Golgi. However, the effective role of CML even in the presence of native LDL suggests CML disrupts the intracellular cholesterol feedback regulation though enhancing the role of SCAP in escorting SREBP-2 from the ER to the Golgi, followed by activating SREBP-2, and upregulating the expression of HMG-CoAR and LDLr, therefore increaseing HMG-CoAR-mediated cholesterol synthesis and LDLr-mediated cholesterol uptake in the renal tubules.

We also evaluated renal function by measuring serum BUN, Cr levels, 24-h urine protein and u-NGAL. Serum Cr level and 24-h urine protein were reduced after AG treatment. U-NGAL was measured to evaluate the tubular function. It is hyperproduced when renal tubules are injury, and is a most promising tubular biomarker in the diagnostic field of diabetic renal tubular disease [34, 35]. In our work, we found AG decreased the u-NGAL level in the diabetic rats. Furthermore, AG alleviated mesangial expansion in the renal glomeruli and basement membrane thickness both in the renal glomeruli and tubules of diabetic rats. The above data suggests inhibiting CML formation improves renal morphology and function in type 2 diabetic rats.

## Conclusions

Taken together, these findings in vivo and in vitro demonstrates that CML disruptes feedback regulation in the diabetic kidney by increasing HMG-CoAR-mediated cholesterol synthesis and LDLr-mediated cholesterol uptake, which cause renal structure and function damage, and ultimately, promotes the development and progress of DN. However, inhibition of CML-induced lipid accumulation might be a potential renoprotective role in DN.

## Abbreviations

AG: Aminoguanidine Hydrochloride
AGEs: Advanced glycation end products
CE: cholesterol ester
CML: Nε-(carboxymethyl) lysine
DN: Diabetic nephropathy
HK-2: human renal tubular epithelial cell line
HMG-CoAR: 3-hydroxy-3-methylclutaryl-CoA reductase
LDLr: low density lipoprotein receptor
PAS: periodic acid Schiff staining
PASM: periodic acid-silver metheramine staining
SCAP: SREBP cleavage-activating protein
SREBP-2: sterol regulatory element binding protein-2
u-NGAL: urinary neutrophil gelatinase-associated lipocalin
